# Delineating meta-quantitative trait loci for anthracnose resistance in common bean (*Phaseolus vulgaris* L.)

**DOI:** 10.3389/fpls.2022.966339

**Published:** 2022-08-25

**Authors:** Safoora Shafi, Dinesh Kumar Saini, Mohd Anwar Khan, Vanya Bawa, Neeraj Choudhary, Waseem Ali Dar, Arun K. Pandey, Rajeev Kumar Varshney, Reyazul Rouf Mir

**Affiliations:** ^1^Division of Genetics and Plant Breeding, Faculty of Agriculture, SKUAST-Kashmir, Wadura, India; ^2^Department of Plant Breeding and Genetics, Punjab Agricultural University, Ludhiana, Punjab, India; ^3^Division of Genetics & Plant Breeding, Faculty of Agriculture, SKUAST-Jammu, Chatha, Jammu and Kashmir, India; ^4^Mountain Agriculture Research and Extension Station, SKUAST-Kashmir, Bandipora, Jammu and Kashmir, India; ^5^College of Life Sciences, China Jiliang University, Hangzhou, China; ^6^State Agricultural Biotechnology Centre, Centre for Crop & Food Innovation, Food Futures Institute, Murdoch University, Murdoch, WA, Australia

**Keywords:** common beans, anthracnose, QTL, meta-QTL, GWAS, candidate genes

## Abstract

Anthracnose, caused by the fungus *Colletotrichum lindemuthianum*, is one of the devastating disease affecting common bean production and productivity worldwide. Several quantitative trait loci (QTLs) for anthracnose resistance have been identified. In order to make use of these QTLs in common bean breeding programs, a detailed meta-QTL (MQTL) analysis has been conducted. For the MQTL analysis, 92 QTLs related to anthracnose disease reported in 18 different earlier studies involving 16 mapping populations were compiled and projected on to the consensus map. This meta-analysis led to the identification of 11 MQTLs (each involving QTLs from at least two different studies) on 06 bean chromosomes and 10 QTL hotspots each involving multiple QTLs from an individual study on 07 chromosomes. The confidence interval (CI) of the identified MQTLs was found 3.51 times lower than the CI of initial QTLs. Marker-trait associations (MTAs) reported in published genome-wide association studies (GWAS) were used to validate nine of the 11 identified MQTLs, with MQTL4.1 overlapping with as many as 40 MTAs. Functional annotation of the 11 MQTL regions revealed 1,251 genes including several R genes (such as those encoding for NBS-LRR domain-containing proteins, protein kinases, etc.) and other defense related genes. The MQTLs, QTL hotspots and the potential candidate genes identified during the present study will prove useful in common bean marker-assisted breeding programs and in basic studies involving fine mapping and cloning of genomic regions associated with anthracnose resistance in common beans.

## Introduction

Common bean (*Phaseolus vulgaris* L.) is an annual legume crop with a relatively small genome size of 473 Mb (Schmutz et al., 2014; Perseguini et al., 2016). It is considered the most important grain legume species in the genus *Phaseolus* for direct human consumption. The species originated in Mexico (Bitocchi et al., 2013) and then spread to South America and gave rise to two distinct gene pools known as Mesoamerican and Andean (Koinange and Gepts, 1992). Dry edible beans supply a major source of quality protein (23%), which is high in lysine and thus complements most cereals. In addition, beans are low in fat content and are packed with complex carbohydrates (55-65%), fiber, vitamins, and minerals (Mitchell et al., 2009; Petry et al., 2015; VazPatto et al., 2015; Vidigal Filho et al., 2020; Jan S. et al., 2021; Choudhary et al., 2022).

Anthracnose (ANT), caused by the hemi-biotrophic fungus *Colletotrichum lindemuthianum* (Sacc. & Magnus), limits the yield potential and drastically affects the quality of seeds and pods of common beans (Sharma et al., 2008; Trabanco et al., 2015; VazBisneta and Gonçalves-Vidigal, 2020). It also reduces the sustainability of crop throughout the world. The pathogen may be responsible for yield losses of up to 65-80% or sometimes 100% if the genotype is highly susceptible and environmental conditions are highly conducive for pathogen (Singh and Schwartz, 2010; Perseguini et al., 2016; Vidigal Filho et al., 2020). To limit the advance of anthracnose in bean crops, integrated strategies are employed that include crop rotation, optimized irrigation and fertilizer, and the use of fungicides. However, these control measures may limit yield potential and increase production costs. The development of resistant cultivars is the most efficient, economical, and environmental friendly strategy to circumvent disease outbreaks (Dillard and Cobb, 1993; Almeida et al., 2021). Therefore, cultivars with different race-specific or broad spectrum resistance genes conferring resistance against multiple races of the pathogens need to be identified and investigated. This enables the use of such cultivars in different common bean breeding programs.

Approximately 25 ANT resistance loci with multiple alleles of resistance from both Mesoamerican and Andean origins have already been identified (Mungalu et al., 2020; Vidigal Filho et al., 2020). For instance, *Co-1* to *Co-17* independent loci harboring resistance have been mapped to the eight chromosomes Pv01, Pv02, Pv03, Pv04, Pv07, Pv08, Pv09, and Pv11 (Zuiderveen et al., 2016). In addition, several other genes have been mapped, including *Co-w, Co-u, Co-z, Co-y*, *CoPv02c*, and *CoPv09c* (Geffroy et al., 2009; Ferreira et al., 2013; Campa et al., 2014). The dominant and monogenic loci of Mesoamerican origin include *Co-2*, *Co-3* (its alleles; *Co-3^2^, Co-3^3^, Co-3^4^*, and *Co-3^5^), Co-4* (its alleles; *Co-4^2^ and Co-4^3^), Co5* (its allele; *Co-5^2^*), *Co-6, Co-11, Co-16, Co-17, Co-u*, and *Co-v*, mapped on chromosomes Pv02, Pv03, Pv04, Pv07, Pv08, and Pv11, respectively (Méndez-Vigo et al., 2005; Geffroy et al., 2008; Gonçalves-Vidigal et al., 2013, 2020; Meziadi et al., 2016). The ANT genes originating from Andean origin are *Co-1* (its alleles; *Co-1^2^, Co-1^3^, Co-1^4^, and Co-1^5^), Co-12, Co-13, Co-14, Co-15, Co-x, Co-w, Co-y, Co-z, Co-Pa, Co-AC, and Co-Pv01CDRK* on chromosomes Pv01, Pv03, and Pv04 (López et al., 2003; Gonçalves-Vidigal and Kelly, 2006; Gonçalves-Vidigal et al., 2013; Richard et al., 2014; Sousa et al., 2015; Coimbra-Gonçalves et al., 2016; Chen et al., 2017; de Lima Castro et al., 2017; Gilio et al., 2020). This diversity causes continuous resistance breakdown in cultivars harboring a single gene, and in certain cases, the particular gene gives resistance exclusively to specific races of the disease, making new races easily overcome the disease (Mahuku and Riascos, 2004).

Numerous QTL mapping studies for ANT disease resistance have also been conducted in different common bean populations (Geffroy et al., 2008; Almeida et al., 2021). In addition, genome-wide association studies (GWAS) for ANT resistance have also been conducted, leading to the identification of a large number of marker-trait associations (MTAs) (Perseguini et al., 2016; Zuiderveen et al., 2016; Wu et al., 2017). We have collected and characterized a set of 428 common bean genotypes from Western Himalayas (Choudhary et al., 2018b) and developed a core set of 96 genotypes (Mir et al., 2021). The core set was also used to better understand the genetic architecture underlying the ANT resistance and interestingly, the study led to the identification of 10 significant MTAs for anthracnose (Choudhary et al., 2018a). Further, identification of more reliable and robust QTLs and refinement of QTL interval without incurring intensive resources is urgently needed to effectively deploy ANT resistance QTLs in breeding programs.

Meta-QTL (MQTL) analysis assembles information from multiple studies and refines QTL locations by narrowing down the confidence intervals obtained from individual studies and correlating them with each other (Goffinet and Gerber, 2000). MQTL analysis involving known QTLs for any particular trait has been conducted in several crops for various traits, including yield-related traits (Tyagi et al., 2015; Saini et al., 2022c), stripe rust resistance (Jan I. et al., 2021), multiple disease resistance (Pal et al., 2022; Saini et al., 2022a), thermo-tolerance (Kumar et al., 2021), salinity stress (Pal et al., 2021), multiple abiotic stress tolerance (Tanin et al., 2022) in wheat, nitrogen use efficiency (Sandhu et al., 2021), grain size and African gall midge resistance (Yao et al., 2016; Daware et al., 2017) in rice and protein and oil content in soybean (Van and McHale, 2017). However, only a very few reports are available for MQTL analysis in this important legume crop for different traits including seed Fe and Zn concentrations (Izquierdo et al., 2018) and white mold resistance (Vasconcellos et al., 2017). However, to the best of our knowledge, MQTL analysis for resistance against ANT in common beans has never been conducted. Therefore, this is the **FIRST** study to report MQTLs for one of the devastating diseases (ANT) in common bean. The objective of the present study was to conduct MQTL analysis for anthracnose followed by the identification of candidate genes (CGs) within the MQTL regions. Efforts were also made to validate the MQTLs with GWAS results and expression analysis of the identified CGs. These MQTLs and CGs will prove useful for marker-assisted breeding (MAB) and for future basic studies to better understand the molecular mechanism of anthracnose resistance in common beans.

## Materials and methods

### Bibliographic review and compilation of quantitative trait loci data

**FIGURE 1:**
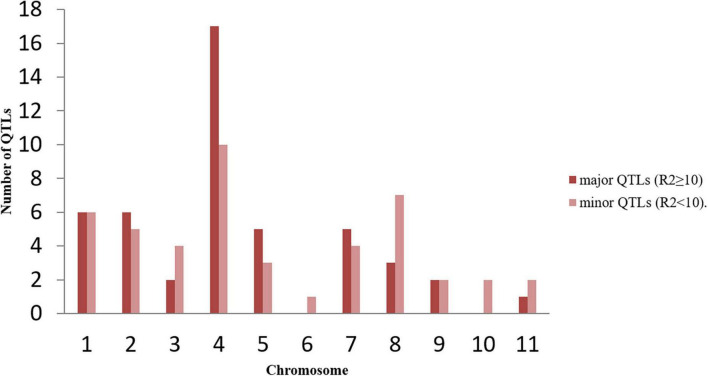
Distribution of major (*R*^2^ ≥ 10) and minor (*R*^2^ < 10) QTLs on different common bean chromosomes used in meta-QTL analysis during the present study. The figure shows maximum number of QTLs are present on chromosome 4.

A detailed bibliographic review was carried out on studies reporting the identification and mapping of QTLs through interval mapping for anthracnose disease resistance in common bean. The database search in Google Scholar^1^ and PubMed^2^ was performed by using the appropriate keywords, *viz.*, common bean, QTL, interval mapping, anthracnose, etc. The QTL information for anthracnose disease resistance in common beans was collected from 18 studies. The information collected included chromosome name, confidence interval (CI), peak position, most closely linked marker(s), LOD score and phenotypic variation explained (PVE or R^2^) for each of the QTL. In cases where the QTL peak position was not available, the average of the CI of the QTL was considered as the peak position of QTL. The R^2^ values of the reported QTLs in the respective studies used in MQTL analysis are represented chromosome-wise in Figure 1. The details on these mapping studies are summarized in Table 1. These previously published studies utilized sixteen different mapping populations which are as follows- Morden003 × OAC Rex (MO), IAC-UNA × CAL 143 (UC), PMB0225 × PHA1037 (PP), BAT93 × JaloEEP558 (BJ), Solwezi × AO-1012-29-3-3A (SA), BRS Estilo × OuroVermelho (BO), B09197 × Nautica (BN), AND-277 × IACMilênio (AM), AmendoimCavalo × PI 207262 (AP), AmendoimCavalo × G2333 (AG), AND 277 × Ouro Negro (AO), Andecha × A493 (AA), California Dark Red Kidney × Yolano (CY), Crioulo 159 × Cornell 49-242 (CC1), Jaguar × Puebla 152 (JP) and Corinthiano × Cornell 49-242 (CC2). All the collected QTLs were given unique identities based on the following information-source study, associated disease resistance trait and chromosome involved.

**TABLE 1:** Details of mapping studies used for anthracnose disease in common bean.

Cross	Population type (size)	Pathotype used	Number of markers used	QTLs identified	References
Morden003/OAC Rex (MO)	F2:3 (182)	Races 73	8	2	Boersma et al. (2013)
IAC-UNA × CAL 143 (UC)	RIL (380)	Race 55	220	15	Oblessuc et al. (2014)
IAC-UNA × CAL 143 (UC)	RIL (150)	Race 38	182	5	Mungalu et al. (2020)
PMB0225 × PHA1037 (PP)	RIL (185)	RACE 23/RACE 1545	229	26	González et al. (2015)
BAT93 × JaloEEP558 (BJ)	RIL (77)	Strain 45, Strain 7,	143	12	Geffroy et al. (2000)
BAT93 × JaloEEP558 (BJ)	RIL (78)	Rwanda/Costa Rica, Tanzania	46	5	Geffroy et al. (2008)
Solwezi × AO-1012-29-3-3A (SA)	RIL (150)	Race 38	182	9	Mungalu et al. (2020)
BRS Estilo × OuroVermelho (BO)	F2 (400)	Lv238, Lv134	28	2	Costa et al. (2021)
B09197 × Nautica (BN)	F2 (94)	Race 73	17	1	Burt et al. (2015)
B09197 × Nautica (BN)	F2:3 (86)	NA	9	1	Chen et al. (2017)
AND-277 × IACMilênio (AM)	BC2F3 (91)	Race 65, Race 81	77	4	Almeida et al. (2021)
AmendoimCavalo × PI 207262 (AP)	F2 (109)	NA	11	1	Gilio et al. (2020)
AmendoimCavalo × G2333 (AG)	F2 (187)	NA	5	1	Gilio et al. (2020)
AND 277 × Ouro Negro (AO)	F2:3 (71)	Race 73	55	1	Gonçalves-Vidigal et al. (2011)
Andecha × A493 (AA)	F2:3 (72)	Race 38	7	1	Méndez-Vigo et al. (2005)
California Dark Red Kidney × Yolano (CY)	F2:3 (71)	NA	84	1	Gonçalves-Vidigal et al. (2020)
Crioulo 159 × Cornell 49-242 (CC1)	F2 (102)	NA	21	3	Coimbra-Gonçalves et al. (2016)
Jaguar × Puebla 152 (JP)	RIL	Race 73	36	1	Zuiderveen et al. (2016)
Corinthiano × Cornell 49–242 (CC2)	F2:3 (86)	NA	23	1	Sousa et al. (2015)

### Construction of consensus map

The high-quality linkage map developed using a large F_2_ population derived from the cross between Stampede × Red Hawk (Song et al., 2015) was downloaded and considered as a reference map which possessed 7,040 markers including 7,015 SNPs. This map currently has the highest density of molecular markers in common bean. The linkage map and the SNPs it contains are expected to make marker-assisted selection and QTL mapping in common bean easier and effective. A consensus genetic map was developed by integrating this high quality reference map with the markers tightly linked to the initial QTLs or flanking the initial QTLs (markers located on the two sides of confidence intervals of individual QTLs) identified from different mapping studies (Supplementary Table 1). For this purpose, LPMerge package of R programming software (Endelman and Plomion, 2014) was utilized.

### Quantitative trait loci projection and meta-analysis

The individual QTL projection on the consensus map was based on their phenotypic variation explained (PVE), LOD score, QTL position, and CI. The QTL projection was performed using QTLProj command available in Biomercator v4.2 software. Following QTL projection, meta-analysis was performed utilizing either of the two different approaches available in the software- (i) single step analysis approach proposed by Goffinet and Gerber (2000) and (ii) two-step analysis approach proposed by Veyrieras et al. (2007). The first approach (Goffinet and Gerber, 2000) was used when the number of projected QTLs per chromosome was 10 or less, and the second approach (Veyrieras et al., 2007) was used when the number of projected QTLs per chromosome was greater than 10. In the first approach, the model with the lowest Akaike Information Criterion (AIC) value was chosen as the best QTL model for ascertaining the number of MQTLs on each chromosome/linkage group. In the second approach, the following five parameters were considered to determine the number of MQTLs on the concerned chromosomes: (i) AIC, (ii) AICc (AIC correction), (iii) AIC3 (AIC 3 candidate models), (iv) BIC (Bayesian information criteria), and (v) AWE (average weight of evidence). The number of MQTLs present on the concerned chromosome was determined by the lowest number of MQTLs predicted in the largest number of models (at least 3 out of these 5).The number of MQTLs on each chromosome (depending on the number of input QTLs on a common genetic map), their consensus positions (based on variance in the positions of input QTLs), and 95% CIs (based on variation in the intervals of input QTLs) were calculated using the chosen model (Sosnowski et al., 2012).

### Identification of the candidate genes and expression analysis

The candidate genes (CGs) available from the MQTL regions were identified based on the position of flanking markers or the markers that are closest to the flanking markers. For this purpose, the most recently annotated version of the *P. vulgaris* reference genome v.2.1 in Phytozome^3^ was used, and the physical positions of the MQTLs and genes contained in these regions were identified. For expression pattern of the identified CGs, a search was made to collect the transcriptomic studies reporting differential expressions of the common bean genes in different plant tissues inoculated with the fungus *C. lindemuthianum*. The collected gene expression datasets were then investigated to ascertain the differential expressions of the CGs underpinning the MQTLs identified in the present study.

### Validation of meta-quantitative trait loci with marker-trait associations identified in earlier genome-wide association studies

To enhance the accuracy of MQTLs identified in the present study, the significant markers or marker-trait associations (MTAs) and promising CGs retrieved from genome-wide association studies (GWAS) were compared to the genomic regions of MQTLs identified for anthracnose resistance in this study. For this purpose, data from 11 different GWA studies on the most stable and significant MTAs/SNPs were collected. These studies included the following: Burt et al. (2015), Perseguini et al. (2016), Zuiderveen et al. (2016), Wu et al. (2017), Fritsche-Neto et al. (2019), Banoo et al. (2020), VazBisneta and Gonçalves-Vidigal, 2020,Vidigal Filho et al. (2020), Almeida et al. (2021), Bisneta et al. (2021) and Costa et al. (2021).

## Results

### Salient characteristics of common bean consensus map

The genetic lengths and number of markers on individual chromosomes revealed substantial variation on the consensus map. The genetic length ranged from 103 cM for chromosome Pv10 to 260 cM for chromosome Pv02. However, number of markers on each chromosome varied from 270 on chromosome Pv06 to 1038 on chromosome Pv11. The consensus map was 2,111.2 cM in length and contained a total of 7,876 markers of various types including, AFLP, RFLP, SSR, SNP, etc. (Supplementary Figure 1). On individual chromosomes, marker density ranged from 1.14 markers per cM on Pv07 to 9.23 markers per cM across Pv11, with a mean of 3.73 markers per cM on the overall genome.

### Meta-quantitative trait loci identified for anthracnose disease resistance

**FIGURE 2:**
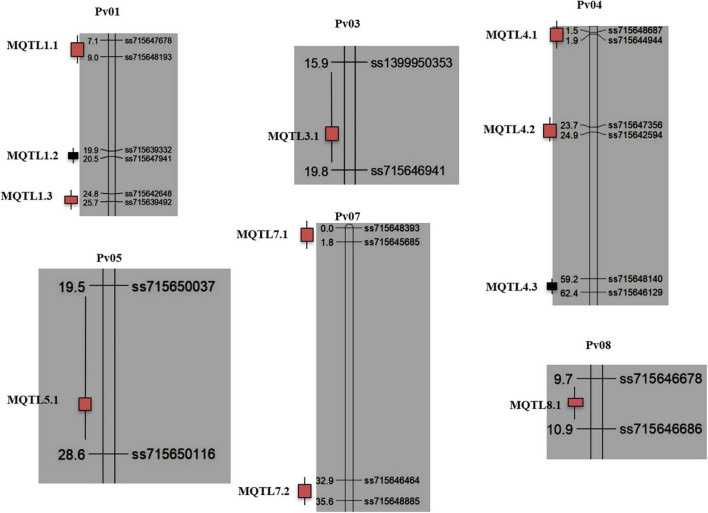
Distribution of 11 MQTLs on 06 common bean chromosomes identified in the study. The boxes on the right side of each chromosome reflect the placements of MQTLs (with the name of the MQTL in each case). The MQTLs highlighted with red color are MQTLs validated through GWAS, while as MQTLs highlighted with black could not be validated. Distances in cM are given to the left of each bar representing a chromosome, and pairs of flanking markers are given to the right of each MQTL.

Of the 92 available QTLs, 88 (95%) QTLs were projected onto the consensus genetic map. Of the 88 QTLs projected onto the consensus map, 14 QTLs remained as single QTLs (Supplementary Table 2), and the remaining 74 QTLs were grouped into 21 potential genomic regions including 11 MQTLs (each involving QTLs from at least two different studies) (Table 2) and 10 QTL hotspots (each involving at least two QTLs identified in an individual study for different traits contributing to resistance). The 11 MQTLs include 3 MQTLs each on chromosomes Pv01 and Pv04, 2 MQTLs on Pv07 and 3 MQTLs each on chromosomes Pv03, Pv05 and Pv08 (Figure 2). The number of clustered QTLs per MQTL ranged from 2 (for 4 MQTLs each located on different chromosomes Pv01, Pv03, Pv05, and Pv07) to 10 (for MQTL4.2 located on chromosome Pv04). LOD score of the individual MQTLs ranged from 2.99 to 83.75 with a mean of 20.38, whereas, phenotypic variation explained by the individual MQTLs ranged from 3.97 to 46.8% with a mean of 16.54%.

**TABLE 2:** Details of 11 Meta-QTLs (MQTLs) identified for anthracnose disease resistance in common beans.

MQTL name	Chr.	Peak position (CI, cM)	Flanking markers	Physical interval (bp)	No. of QTLs involved	*QTLs involved	LOD score (PVE value)
MQTL1.1	1	8.03 (7.09–8.97)	ss715647678–ss715648193	2691475–3444171	4	Geffroy, 2008_DS_ANT2_1, Geffroy, 2008_DS_ANT1_1, Gilio, 2020b_DS_ANT2_1, Gilio, 2020a_DS_ANT1_1	3.72 (7.52)
MQTL1.2	1	20.2 (19.85–20.54)	ss715639332–ss715647941	7026852–8046958	2	Zuiderveen, 2016_DR_ANT1_1, Geffroy, 2000_DS_ANT7_1	20.72 (46.8)
MQTL1.3	1	25.26 (24.79–25.73)	ss715642648–ss715639492	17159280–39410152	3	González, 2015_AUDPC_ANT11_1, González, 2015_AUDPC_ANT9_1, Gonçalves -Vidigal2020_DS_ANT1_1	7.84 (6.87)
MQTL3.1	3	17.86 (15.94–19.78)	ss1399950353–ss715646941	2121717–2620445	2	Oblessuc, 2014_DS_ANT2_2, Geffroy, 2000_DS_ANT2_3	2.99 (8.85)
MQTL4.1	4	1.68 (1.51–1.85)	ss715648687–ss715644944	328788–1638581	4	Méndez-Vigo et al., 2005_DS_ANT1_4, Costa, 2021_DS_ANT1_4, Coimbra-Gonçalves, 2016_DS_ANT1_4, Costa, 2021_DS_ANT2_4	3 (23.26)
MQTL4.2	4	24.33 (23.72–24.94)	ss715647356–ss715642594	45349844-45414255	10	González, 2015_AUDPC_ANT6_4, Geffroy, 2000_DS_ANT8_4, Boersma, 2013_DR_ANT2_4, Geffroy, 2000_DS_ANT3_4, González, 2015_LAUDPC_ANT21_4, Chen, 2017_DR_ANT1_4, Boersma, 2013_DR_ANT1_4, González, 2015_LDC_ANT15_1, González, 2015_SDC_ANT2_4, Coimbra-Gonçalves, 2016_DS_ANT2_4	83.75 (23.2)
MQTL4.3	4	60.76 (59.15–62.36)	ss715648140–ss715646129	41929814–42755828	5	Coimbra-Gonçalves, 2016_DS_ANT3_4, Sousa, 2015_DR_ANT1_4, Geffroy, 2000_DS_ANT4_4, Geffroy, 2008_DS_ANT3_4, Geffroy, 2008_DS_ANT5_4	14.57 (23.2)
MQTL5.1	5	24.04 (19.47–28.61)	ss715650037–ss715650116	1150129–2501072	2	Almeida, 2021_DS_ANT1_3, González, 2015_LAUDPC_ANT22_5	7.85 (6.74)
MQTL7.1	7	0.45 (0–1.78)	ss715648393–ss715645685	61520–606814	4	Geffroy, 2000_DS_ANT9_7, González _LDC_ANT19_7, González, 2015_LAUDPC_ANT25_7, Geffroy, 2000_DS_ANT5_7	29.15 (10.03)
MQTL7.2	7	34.27 (32.93–35.62)	ss715646464–ss715648885	4144345–4742127	2	Mungalu, 2020_DS_ANT13_7, Geffroy, 2000_DS_ANT10_7	41.12 (21.55)
MQTL8.1	8	10.26 (9.67–10.85)	ss715646678–ss715646686	392355–569881	5	González, 2015_SDC_ANT5_8, Oblessuc, 2014_DS_ANT10_8, González, 2015_LDC_ANT20_8, González, 2015_LAUDPC_ANT26_8, González, 2015_AUDPC_ANT8_8	9.55 (3.97)

chr., chromosome, CI, confidence interval, LOD, Logarithm of Odds, PVE, phenotypic variation explained; *unique identities given to the initial QTLs based on source study, associated disease resistance trait (DS, disease severity, DR, disease resistance, AUDPC, area under disease progress curve, SDC, stem disease score, LAUDPC, leaf area under disease progress curve, LDC, leaf disease score), ANT, anthracnose and chromosome number.

Furthermore, 10 genomic regions that involved at least two initial QTLs from the same study were also identified. These QTLs can be considered as QTL hotspots and include 3 QTL hotspots on Pv02, 2 QTL hotspots on Pv09, and each on chromosomes Pv03, Pv04, Pv05, Pv07, and Pv08 (Table 3). The chromosome-wise CI of the identified MQTLs was lower than that of their initial QTLs and ranged from 0.34 to 20 cM. However, the chromosome-wise average CI (95%) for the initial QTLs in interval mapping studies ranged from 2.28 to 28.50 cM. The average CI of the identified MQTLs (2.44) was 3.51 fold less than that of average CI of the initial QTLs (8.57). The identified MQTLs are discussed as follows-

**TABLE 3:** Details of quantitative trait loci (QTL) hotspots identified for anthracnose disease resistance in common beans.

QTL name	Chr.	Peak position (CI, cM)	Flanking markers	Physical interval (bp)	No. of QTLs involved	*QTLs involved
QTL_hotspot2.1	2	19.4 (16.9–21.86)	ss715648913–ss715644429	17959594–43001788	2	Geffroy, 2008_DS_ANT4_2, Geffroy, 2008_DS_ANT4_2
QTL_hotspot2.2	2	35.3 (33.6–37.07)	ss715646371–ss715639502	2794926–3118774	6	Mungalu, 2020_DS_ANT4_2, Mungalu, 2020_DS_ANT2_2, Mungalu, 2020_DS_ANT3_2, Mungalu, 2020_DS_ANT3_2, Mungalu, 2020_DS_ANT5_2, Mungalu, 2020_DS_ANT1_2
QTL_hotspot2.3	2	63.1 (60.3–66)	ss715639428–BM172	28656450–45866753	2	Oblessuc, 2014_DS_ANT3_, Oblessuc, 2014_DS_ANT2_2
QTL_hotspot3.1	3	47 (46–48.05)	ss715640761–ss715641926	21279773–30433310	2	González, 2015_LDC_ANT14_3, González, 2015_AUDPC_ANT12_3
QTL_hotspot4.1	4	45.4 (44.9–45.89)	ss715650687–ss715639504	35906204–37518852	6	Mungalu, 2020_DS_ANT8_4, Mungalu, 2020_DS_ANT12_4, Mungalu, 2020_DS_ANT9_4, Mungalu, 2020_DS_ANT7_4, Mungalu, 2020_DS_ANT10_4, Mungalu, 2020_DS_ANT11_4
QTL_hotspot5.1	5	36.9 (36.3–37.5)	ss715650071–ss715647940	3458507–3818284	5	González, 2015_LAUDPC_ANT24_5, González, 2015_SDC_ANT4_5, González, 2015_LDC_ANT18_5, González, 2015_AUDPC_ANT13_5, González, 2015_AUDPC_ANT7_5
QTL_hotspot7.1	7	94.7 (91.5–97.86)	ss715645173–ss715645220	48372720–51176264	2	Oblessuc, 2014_DS_ANT8_7, Oblessuc, 2014_DS_ANT7_7
QTL_hotspot8.1	8	2.44 (1.64–3.25)	SC00089in640327_372918–sc00101ln599358_4090	497385–5150618	2	Burt, 2015_DR_ANT2_8, Burt, 2015_DR_ANT1_8
QTL_hotspot9.1	9	0 (0–4.77)	ss715644148–ss715645103	128333–8749314	2	Oblessuc, 2014_DS_ANT13_9, Oblessuc, 2014_DS_ANT14_9
QTL_hotspot9.2	9	19.8 (17.4–22.26)	ss715646560–ss715645165	12400878–14425028	2	González _LDC_ANT17_9, González, 2015_LAUDPC_ANT23_9

chr., chromosome, CI, confidence interval, *unique identities given to the initial QTLs based on source study, associated disease resistance trait (DS, disease severity, DR, disease resistance, AUDPC, area under disease progress curve, SDC, stem disease score, LAUDPC, leaf area under disease progress curve, LDC, leaf disease score), ANT, anthracnose and chromosome number.

### Meta-quantitative trait loci on chromosome Pv01

The three MQTLs detected on chromosome Pv01 include the following- MQTL1.1, MQTL1.2 and MQTL1.3 (Figure 2). MQTL1.1 comprises four initial QTLs identified from three common bean mapping populations, BJ, AJ and AO, conferring resistance to isolates of strain 45, strain 7 and Rwanda/Costa Rica and Tanzania, local isolates. The initial QTLs involved two closely related genes, *Co-x* and *Co-w*, from the Andean source (JaloEEP558) and *Co-AC* from Amendoim Cavalo (AC), which also belongs to the Andean gene pool and is resistant to 13 races including seven Mesoamerican (9, 65, 73, 89, 1545, 2047, and 3481) and six Andean (2, 7, 19, 23, 39, and 55) races. MQTL1.2 also comprises QTLs identified from the BJ mapping population and the *Co-1* gene from another Mesoamerican RIL population, JP. MQTL1.3 comprises three initial QTLs, two from the AO and Andean mapping populations and one from the CY (Andean × Mesoamerican) mapping population. The two QTLs from the AO population had significant effects on stem resistance against races 23 and 1545.

### Meta-quantitative trait loci on chromosome Pv03

The MQTL3.1 was identified on chromosome Pv03 (Figure 2) and it comprises the two initial QTLs from UC and BJ mapping populations. The QTL from BJ had stem resistance (with 82% PVE), and the alleles for increased resistance were inherited from the parent JaloEEP558.

### Meta-quantitative trait loci on chromosome Pv04

The three MQTLs identified on chromosome Pv04 include MQTL4.1, MQTL4.2, and MQTL4.3 (Figure 2). The MQTL4.1 comprises 4 initial QTLs derived from three mapping populations (BO, AA, and CC1). The two initial QTLs from the BO mapping population showed resistance to isolates Lv138 and Lv238, respectively. The other initial QTL from the AA mapping population had resistant alleles contributed from the A493 parent conferring resistance to race 38 and race 6. Furthermore, the initial QTL from the CC1 mapping population is co-localized with the *Co-16* gene and is inherited from the parent Crioulo 159, which provides anthracnose resistance to race 2047. The MQTL4.2 comprised the 10 initial QTLs, making it the MQTL with the largest number of clustered QTLs from 5 common bean mapping populations (MO, BJ, PP, BN, and CC1). Two initial QTLs from the MO mapping population included two R genes (from parent Morden003 resistant to race 73 and race 105). Similarly, the initial QTLs from the BJ population conferred resistance for stems and leaves against strain 7 and strain 45, whereas, four main effects initial QTLs from the PP mapping population had significant effects on stem and leaf resistance against race 23. The MQTL4.3 comprised 5 initial QTLs from three mapping populations (CC1, CC2 and BJ). The CC1 mapping population had the *Co-15* gene from the Andean bean cultivar Corinthiano, which confers resistance to race 2047. The CC2 mapping population also had the same gene, *Co-15*, conferring resistance to race 2047. The other three initial QTLs from the BJ mapping population included genes for stem, petiole and leaf resistance on Pv04 against strain 7 and strain 45.

### Meta-quantitative trait loci on chromosome Pv05

The MQTL5.1 was identified on chromosome Pv05 (Figure 2) and it comprised of two initial QTLs from two mapping populations (AM and PP). The AM population has resistance to races 64, 65, 73, 81, 87, 89, 119, 453, and 2047. The resistance gene was contributed from the parent AND-277. The initial QTL from the PP mapping population conferred leaf resistance against race 1545.

### Meta-quantitative trait loci on chromosome Pv07

Two MQTLs were identified on chromosome Pv07, including MQTL7.1 and MQTL7.2 (Figure 2). The MQTL7.1 comprised 4 initial QTLs, two from BJ and two from the PP mapping population. Two genomic regions from the BJ mapping population were associated with both stem and petiole resistance against strain 7 and strain 45. However, initial QTLs from the PP mapping population had leaf resistance against race 1545. The MQTL7.2 comprised two initial QTLs from two mapping populations (SA and BJ). The initial QTL from the SA mapping population provided resistance to race 39, with PVE 22.1%. The resistant allele was contributed from the parent AO-1012-29-3-3A. Another QTL from the BJ mapping population was associated with stem and petiole resistance against strain 7 and strain 45.

### Meta-quantitative trait loci on chromosome Pv08

The MQTL8.1 was identified on chromosome Pv08 (Figure 2) and comprised four initial QTLs from the PP mapping population and one initial QTL from the UC mapping population. The four initial QTLs from the PP mapping population were the major effect QTLs having stem and leaf resistance to race 1545. The initial QTL from UC was mapped for race 4.

### Candidate gene identification and expression analysis

**FIGURE 3:**
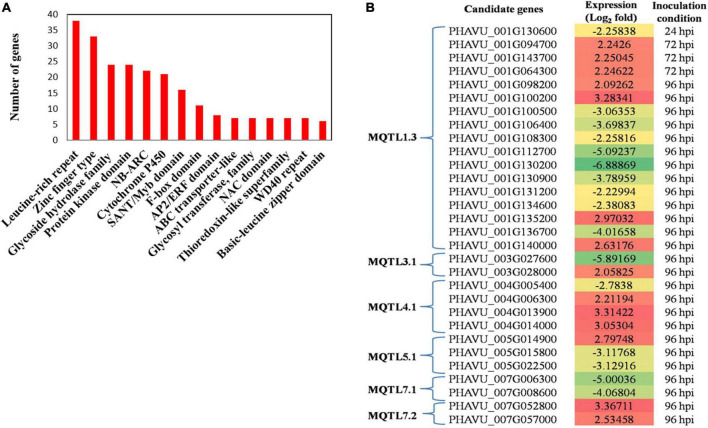
**(A)** The distribution of number of candidate genes encoding different proteins associated with disease resistance traits. **(B)** Heat map showing differential expression of proteins encoded by candidate genes associated with MQTLs at different hours post inoculation (hpi) (FC = 3 to -6).

In the present study, 302 candidate genes were selected from the total of 1,251 candidate genes (Supplementary Table 3) available from the MQTL regions. These CGs were selected based on their function related to disease resistance and related traits. They belonged to the 11 MQTLs as follows: MQTL1.1 (12), MQTL1.2 (12), MQTL1.3 (127), MQTL3.1 (10), MQTL4.1 (61), MQTL4.2 (4), MQTL4.3 (19), MQTL5.1 (27), MQTL7.1 (20), MQTL7.2 (8) and MQTL8.1 (2). Most of the CGs belongs to leucine-rich repeat (LRR) proteins associated with innate immunity in plants and zinc finger proteins, which are the largest known DNA-binding protein family. Other major identified CGs belong to the protein kinase domain, NBS-ARC domain-containing protein (functional ATPase domain, and its nucleotide-binding state is proposed to regulate the activity of the R protein), cytochrome P450 and glycoside hydrolase family (Figure 3A). The differentially expressed genes (DEGs) associated with changes in plant gene expression over the course of infection have been identified using transcriptome analysis in the common beans- *C. lindemuthianum* system (Padder et al., 2016). Out of the 302 CGs characterized in the study, 30 CGs (Table 4) were common to the DEGs identified in the study conducted by Padder et al. (2016). Interestingly, 30 CGs were found to be associated with 07 of the 11 MQTLs, with a maximum of 16 CGs associated with MQTL1.3. These CGs can be considered the more promising CGs for pathogenicity and disease-related traits in common beans (for details see Figure 3B and Table 4).

**TABLE 4:** List of candidate genes identified within the physical intervals of Meta-QTLs (MQTLs) validated through differential expression.

MQTL	Gene stable ID	Gene start (bp)	Gene end (bp)	Interpro description
MQTL1.2	*PHAVU_001G064300g*	8039774	8042757	SWEET sugar transporter^a^
MQTL1.3	*PHAVU_001G094700g*	20564994	20570123	CBS domain^d^
MQTL1.3	*PHAVU_001G098200g*	21769115	21770842	Glycoside hydrolase family 16^a^
MQTL1.3	*PHAVU_001G100200g*	23040328	23042480	NAC domain^b^
MQTL1.3	*PHAVU_001G100500g*	23149740	23152402	NAC domain^b^
MQTL1.3	*PHAVU_001G106400g*	27221418	27224141	Zinc finger, DOF-type^b^
MQTL1.3	*PHAVU_001G108300g*	28331083	28333279	Multi antimicrobial extrusion protein^b^
MQTL1.3	*PHAVU_001G112700g*	31425398	31429114	Glycoside hydrolase, family 3, N-terminalb
MQTL1.3	*PHAVU_001G130200g*	36911989	36914461	Glycosyltranferase 61^b^
MQTL1.3	*PHAVU_001G130600g*	36977445	36977753	ABC transporter-like^c^
MQTL1.3	*PHAVU_001G130900g*	37058999	37061900	ABC transporter-like^b^
MQTL1.3	*PHAVU_001G131200g*	37161671	37179976	ABC transporter-like^b^
MQTL1.3	*PHAVU_001G134600g*	37948400	37952635	Protein kinase domain^a^
MQTL1.3	*PHAVU_001G135200g*	38007144	38012525	Glycosyl hydrolases 36^b^
MQTL1.3	*PHAVU_001G136700g*	38316315	38318940	Zinc finger C2H2-type^a^
MQTL1.3	*PHAVU_001G140000g*	38799607	38800605	Leucine-rich repeat^a^
MQTL1.3	*PHAVU_001G143700g*	39366107	39367905	Thioredoxin domain^b^
MQTL3.1	*PHAVU_003G027600g*	2550469	2556320	Protein kinase domain^a^
MQTL3.1	*PHAVU_003G028000g*	2619872	2622387	SANT/Myb domain^b^
MQTL4.1	*PHAVU_004G005400g*	395119	396899	Cytochrome P450^a^
MQTL4.1	*PHAVU_004G006300g*	489427	490938	Cytochrome P450^a^
MQTL4.1	*PHAVU_004G013900g*	1461022	1464245	GDSL lipase/esterase^a^
MQTL4.1	*PHAVU_004G014000g*	1467502	1471313	Tetratricopeptide-like helical domain superfamilya
MQTL5.1	*PHAVU_005G014900g*	1335454	1339316	Protein kinase domain^a^
MQTL5.1	PHAVU_005G015800g	1396464	1401960	Cytochrome P450^b^
MQTL5.1	PHAVU_005G022500g	2000935	2005462	Glycoside hydrolase, family 28^b^
MQTL7.1	PHAVU_007G006300g	455903	459115	Leucine-rich repeat^d^
MQTL7.1	PHAVU_007G008600g	599228	601518	Chalconeisomerase^b^
MQTL7.2	PHAVU_007G052800g	4246064	4248328	Glycoside hydrolase family 16^a^
MQTL7.2	PHAVU_007G057000g	4728529	4732414	Protein kinase domain^a^

a, up-regulation in resistant lines at 96 hpi; b, up-regulation in susceptible lines at 96 hpi; c, up-regulation in susceptible line at 24 hpi; d, up-regulation in resistant line at 72 hpi; (hpi, hours post inoculation).

### Meta-quantitative trait loci validated through earlier genome-wide association studies

**FIGURE 4:**
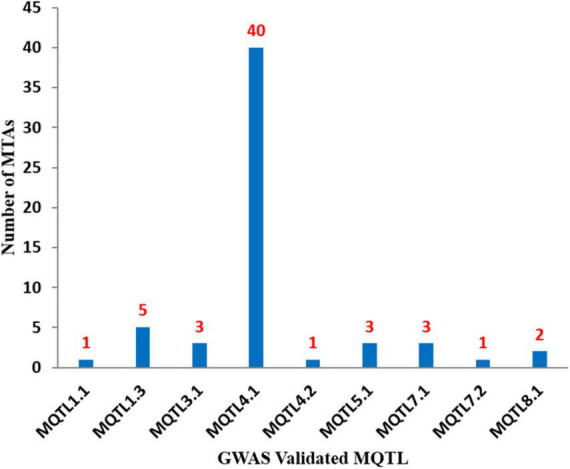
Number of known GWAS based MTAs which validated each of 9 MQTLs identified during the present study. The figure shows maximum number of MTAs validated for MQTL4.1.

The physical positions of the MQTLs identified in the present study were also compared with MTAs and promising CGs reported in previous GWA studies. Among the 11 MQTLs reported in the present study, 9 MQTLs co-localized with at least one MTA available from the GWAS. A total of 59 MTAs were identified from 09 different GWA studies for anthracnose in common beans (Figure 4 and Supplementary Table 4). Some of the MQTLs were found to be co-localized with MTAs available from more than one GWA study, for instance, MQTL4.1 co-localized with 40 MTAs reported in eight different GWA studies (Zuiderveen et al., 2016; Wu et al., 2017; Banoo et al., 2020; VazBisneta and Gonçalves-Vidigal, 2020; Vidigal Filho et al., 2020; Almeida et al., 2021; Bisneta et al., 2021; Costa et al., 2021). Similarly, MQTL1.3 co-localized with 06 MTAs reported in five different GWA studies (Perseguini et al., 2016; Wu et al., 2017; Banoo et al., 2020; VazBisneta and Gonçalves-Vidigal, 2020; Vidigal Filho et al., 2020). Further, several promising CGs identified through GWAS were also found to be co-localized with some of the MQTLs identified during the present study, for instance, MQTL1.3 included *Phvul.001G098100*, *Phvul.001G128200.1*, *Phvul.001G1332000*, and *Phvul.001G141000*; MQTL4.1 co-localized with *Phvul.004G005800*, *Phvul.004G006300*, *Phvul.004G006800*, *Phvul.004G012600*, and *Phvul.004G012801*; MQTL5A.1 overlapped with *Phvul.005G020300.1*; and MQTL7.1 included *Phvul.007G006200*.

## Discussion

To get a deeper knowledge of the genetic architecture underlying anthracnose resistance in common bean and to identify the most important hot-spot genomic regions for this disease, we used meta-QTL analysis of previously reported QTLs retrieved from different genetic mapping studies. In total, 92 QTLs identified using 16 mapping populations in 18 previous QTL mapping studies were projected onto the consensus linkage map constructed in the present study. Out of the 88 projected QTLs, 74 were grouped into 21 potential genomic regions including 11 MQTLs and 10 QTL hotspots, suggesting that most of the previously mapped anthracnose resistance loci were shared by multiple populations and/or exhibited resistance to multiple isolates/races. This study is the first meta-analysis where more than 95% of the QTLs could be projected on a consensus map. The consensus map developed had average marker density of 3.73 markers per cM. The density of markers was significantly larger near the fore-end of the chromosome. These results were in agreement with several earlier studies (e.g., Liu et al., 2020; Yang et al., 2021; Saini et al., 2022a). This was mostly because the independent genetic maps that were utilized to create the consensus map contained different types and numbers of markers, but ultimately, it was the best consensus map that could be created with a lot of marker data. A meta-QTL analysis for another important disease white mold resistance was conducted earlier in common bean (Vasconcellos et al., 2017). In this study, 37 QTLs identified across 14 RIL populations were clustered into 17 MQTLs for white mold resistance. Nine of these 17 MQTLs had confidence intervals ranging from 0.65 to 9.41 Mb (Vasconcellos et al., 2017). Similarly, Klein et al. (2020) utilized 17 QTLs for seed weight per plant, 16 QTLs for seed number per plant, 35 QTLs for thousand seed weight, and 21 QTLs for seed protein content and identified 27 MQTLs associated with different traits in pea. Small CIs of less than 2 cM were found in several of them, covering less than a hundred underlying candidate genes.

All the genomic regions associated with ANT resistance investigated in the present study can be grouped into two major groups: (i) ***Stable and perfect MQTLs under different environments***: One of the important features of QTLs that could fit in MAS is stability across a wide range of environments. A total of 11 MQTLs were identified during the present study. For instance, MQTL4.2 is based on 10 initial QTLs from five different studies (Geffroy et al., 2000, Boersma et al., 2013, González et al., 2015; Coimbra-Gonçalves et al., 2016). Similarly, MQTL4.3 and MQTL8.1 are based on 5 initial QTLs from three and two different studies, respectively. Similar results have also been described by various studies for clustering of different initial QTLs into a MQTL (Klein et al., 2020; Liu et al., 2020; Kaur et al., 2021; Gudi et al., 2022). Most of the MQTLs identified in this study had a reasonably narrow CI, resulting in markers that are more strongly and closely linked to the corresponding MQTL (Figure 2). The longer CI for a few MQTLs may be due to unusual recombination in that particular region or any other reason which may be studied in further studies. Using an overlap region of four MQTLs for yield-related traits on chromosome A05 of peanut, a region of the map that previously co-localized multiple major QTLs for pod traits was narrowed from 3.7 to 0.7 cM (Lu et al., 2018). Furthermore, 493 QTLs associated with different diseases such as septoria tritici blotch, septoria nodorum blotch, fusarium head blight, karnal bunt, and loose smut identified in 59 different mapping studies were utilized for the prediction of 63 MQTLs for disease resistance, 11 of these MQTLs conferred resistance to more than 3 diseases in wheat Saini et al. (2022a).

Overall, four of the 11 MQTLs are considered as most promising MQTLs which include: MQTL1.3 having 06 MTAs from 5 different GWA studies and 16 CGs validated from the expression analysis, MQTL4.1 with 40 CGs validated from eight distinct GWA studies, MQTL4.2 having highest LOD value and included 10 initial QTLs from 5 different mapping populations and MQTL1.2 with highest phenotypic variation explained (*R*^2^ = 46.8). SNP markers closely associated with either of the above MQTLs may be selected and converted to Breeder-friendly Kompetitive allele-specific polymorphic chain reaction (KASP) markers (Kaur et al., 2020) utilizing any available software and may be validated using an appropriate population developed from the two contrasting parental lines.

(ii) ***QTL hotspots:*** A total of 10 QTL hotspots that involved more than one QTL from the same study were also identified. These QTLs can be considered unique QTLs as they did not cluster with the QTLs identified from other mapping populations. These QTL hotspots can also be considered important for future breeding programs aimed at enhancing anthracnose resistance in common bean utilizing resistant parent of the corresponding mapping populations as the ‘donor parent’.

### Insights into the genetic architecture underlying the anthracnose disease resistance

Several genes conferring resistance to anthracnose have already been identified on different chromosomes of common bean. Chromosome Pv01 harbors various known resistance genes and their alleles (*Co-1*, *Co-1^2^*, *Co-1^3^*, *Co-x*, *Co-w* and *Co-u*) for different races of anthracnose (Zuiderveen et al., 2016). Among them, *Co-x* and *Co-w* are two closely linked genes from the Andean JaloEEP558 genotype that are mapped at the end of chromosome Pv01 (Geffroy et al., 2008). Combining Andean and Meso-american resistance genes is an important approach to obtain durable resistance against anthracnose in common beans. In this study, MQTL1.1 had the *Co-x* gene from the BJ (Meso-American × Andean), *Co-AC* from AP (Andean × Carioca) and *Co-1* gene from the JP mapping population. This gene *Co-x* confers resistance to strains 100, E25, 3616, and 82 and thus has great potential for use in the development of mapping populations (Pastor-Corrales et al., 1994; Geffroy et al., 2008). Furthermore, the *Co-AC* gene was positioned downstream of the *Co-1* allele (75.8 Kbp) by using an RIL population (Jaguar × Puebla 152) (Geffroy et al., 2008). These results suggest that the *Co-AC* locus is different from that of *Co-x, Co-1* and its alleles.

Furthermore, MQTLs identified on chromosome Pv04 involve 19 initial QTLs. They include important initial QTLs such as MQTL4.1 included *Co-3/Co-9, Co-16*, MQTL4.2 included *Co-3^4^* and MQTL4.3 included *Co-15*. For MQTL4.1, two initial QTLs from the BO mapping population were positioned between 0.0111 and 0.2270 Mb and 1.1345 Mb and 1.1657 Mb, respectively, and showed resistance to isolates Lv138 and Lv238, respectively (Costa et al., 2021). Furthermore, the initial QTL from the CC1 mapping population is co-localized with the *Co-16* gene and is inherited from the parent Crioulo 159, which provides anthracnose resistance to race 2047. Geffroy et al. (1999) indicated that *Co-9* and *Co-y/Co-z* are formed by an R gene cluster and are alleles at a complex locus located on chromosome Pv04. It has also been described that *Co-3* and *Co-9* are alleles of the same gene (Méndez-Vigo et al., 2005). Geffroy et al. (2008) described the *Co-3^3^* allele in the differential cultivar BAT 93 and mapped it on Pv04. *Co-15* is another independent locus located on a distinct region of chromosome Pv04 and provides resistance to the race 2047 (Sousa et al., 2015). The other three initial QTLs included the genes *Co-y* and *Co-z* from the Andean parent (JaloEEP558) and *Co-9* from the Mesoamerican parent (BAT93). In addition, MQTL1.3, MQTL4.2, MQTL5.1, MQTL7.1, and MQTL8.1 have main effect initial QTLs from a common source of mapping population developed between two Andean lines of common bean (PMB0225 x PHA1037). These initial QTLs confer organ-specific resistance to race 23 and race 1545, including stem, leaf, and petiole resistance (González et al., 2015). Gene pyramiding is a crucial approach for creating the most stable genotypes with long-term resilience in a wide range of environments (Dormatey et al., 2020). As a result, the MQTLs discovered in this study can be employed in breeding programs that incorporate Andean and Mesoamerican genes to confer resistance to virulent races of *C. lindemuthianum*.

In addition, in the present study, different sources of resistance, such as BAT93 (carrying *Co-3*), JaloEEP558 (*Co-x* and *Co-w*), Amendoim Cavalo (*Co-AC*), A493 (*Co-3/Co-9*), Crioulo 159 (*Co-16*), Morden003 (two R gene loci; R73 and R105), and Corinthiano (*Co-15*) utilized for the development of different mapping populations were considered (Table 1). For instance, MQTL1.1, MQTL1.2, MQTL3.1, MQTL4.2, MQTL4.3 and MQTL7.2 had initial QTLs from one common source (BJ mapping population) and several other sources. This shows that although these sources share certain genes for partial anthracnose resistance, they may have distinct alleles for some of the loci. The various alleles that provide partial resistance could be valuable in identifying alleles at common loci that have less linkage drag from surrounding harmful genes and/or conditioning the highest level of resistance. This could be accomplished by using marker-assisted backcrossing to create near-isogenic lines (NILs) with distinct alleles from a common recurrent parent. However, it would be beneficial from a breeding standpoint to select certain resistance sources that are best suited to the genetic background one is attempting to improve. Some of the earlier meta-QTL studies conducted in common bean also involved the use of several populations, for instance, 14 RIL populations for white mold resistance (Vasconcellos et al., 2017) and seven mapping populations, including two Andean, two Middle American and three intergene pool populations for discovery of meta-QTLs for seed Fe and Zn content (Izquierdo et al., 2018).

### Candidate gene identification and expression analysis

Meta-QTLs (MQTLs) are thought to be potential targets for identifying candidate genes (CGs) linked to the traits of interest in crop plants. Various meta-analysis studies have demonstrated that MQTL regions have a strong association with the density of genes in a genome, such as in wheat and maize (Swamy et al., 2011; Quraishi et al., 2017; Saini et al., 2021). Several CGs underlying MQTLs have been reported in legumes, including those for seed productivity and quality parameters in pea (Klein et al., 2020), amino acid, protein, and oil content in soybean (Van and McHale, 2017; Gong et al., 2018), and seed size in cowpea (Lo et al., 2019). CGs underpinning MQTLs were also reported by us during the present study by utilizing the common bean genomic sequences available at Phytozome (see text footnote 3). An extensive review of the literature revealed their involvement in a variety of functions including membrane integrity, ADP/ATP binding, defense response, transcription and translational regulation of various genes, nucleic acid, protein, and metal ion binding. In the present study, genes encoding for NBS-LRR domains containing proteins were identified from different MQTL regions, including MQTL1.3, MQTL3.1, MQTL4.1, MQTL4.3 and MQTL7.1. NBS-LRRs recognize particular pathogen-encoded effector proteins. When activated, NBS-LRRs usually trigger the hypersensitive response, a type of localized programmed cell death (PCD) that is thought to aid resistance by isolating the infection physically (Heath, 2000; Padmanabhan et al., 2009). In peanut, one main effect MQTL was identified for late leaf spot resistance at 0.38 cM. In this region, BLAST searches discovered 26 CGs, some of which were annotated as linked to disease resistance regulation in several plant species (Lu et al., 2018).

Overall, the present study identified at least 302 CGs (Supplementary Table 3) encoding different proteins involving the following: ABC transporter type 1, trans membrane domain, AP2/ERF domain-containing proteins, basic-leucine zipper domain-containing proteins, calcium-dependent channel, cytochrome P450, glycoside hydrolase family domain-containing proteins, protein kinase, NAC transcription factor, NB-ARC domain, SANT/Myb domain, zinc finger domain containing proteins, etc. Recent findings suggest that these domains are important in R protein interactions as well as in other defense related proteins including pathogen effector proteins and in the activation of innate immunity signal transduction pathways (Liu et al., 2007; Kumar et al., 2020; Jan I. et al., 2021). For instance, the rice *Xa21* gene for bacterial blight resistance (*Xanthomonas*) and the tomato *Cf* genes for resistance to the fungal pathogen *Cladosporium fulvum* belong to the extracellular LRR class of R genes (Ellis et al., 2000). Promising CGs such as ethylene responsive and MYB transcription factors, cell wall receptor kinase, and peroxidase having differential expression under *Sclerotinia sclerotiorum* infection were found within the narrower CI for five of the nine MQTLs in a study conducted by Vasconcellos et al. (2017) and Jan I. et al. (2021) also discovered important CGs underlying MQTLs for stripe rust in wheat, such as those encoding NBS-LRR proteins, UDP-glucosyltransferases, WRKY proteins, transporters and MAP kinases, which could correspond to known *Yr* genes or be involved in downstream signaling processes during the wheat-*Puccinia striiformis* interaction.

On the basis of significant changes in gene expression, (fold change -6 to + 3), 30 CGs encoding proteins involved in providing disease resistance were also detected (for further details, see Figure 3B and Table 4); these genes can be subjected to further analyses for a better understanding of the molecular mechanism underlying anthracnose resistance as well as for the development of markers for breeding disease-resistant common bean cultivars. Up-regulation of resistance genes on Pv01 harboring MQTL1.3 containing LRR (*PHAVU_001G134600*) and NAC domain containing protein (*PHAVU_001G100500*) strongly favors resistance against *C*. *lindemuthianum*. Pv01 contains *Co-1* and *Co-x* genes. The *Co-x* gene had eight putative genes within the 58 kb region, and two kinases and one cytochrome P450 showing up regulation (Padder et al., 2016). Up-regulation of resistance genes on Pv01 with NB-ARC and LRR domains, especially *Hs1^Pro–1^*(*Phvul.001G241300*), enhances *C. lindemuthianum* effector interactions with these genes. *Hs1^Pro–^*^1^ has a putative transmembrane domain and is known to provide cyst nematode resistance in sugar beets on a gene-for-gene basis (Cai et al., 1997). Similarly, different meta-QTL studies have identified promising CGs associated with MQTLs with differential expression patterns for the traits under studies, such as drought tolerance in wheat (Kumar et al., 2020), root traits in wheat (Saini et al., 2021) and yield-related traits and seed protein content in pea (Klein et al., 2020).

### Validating meta-quantitative trait loci from genome-wide association studies

Genome-wide association studies (GWAS) is one of the most widely used methods of gene discovery nowadays and in use in almost all important crop plants (e.g., Choudhary et al., 2018b; Sidhu et al., 2020; AlTameemi et al., 2021). This method allows for high-resolution mapping of genes/QTLs utilizing recent as well as historical recombination events in natural populations (Saini et al., 2022b). In the present study, 59 MTAs from 10 previously known GWA studies were identified to be co-localized with 09 of the 11 MQTLs (Supplementary Table 4). As many as 40 MTAs derived from 8 distinct GWA studies were co-localized with MQTL4.1. More interestingly, MQTL1.3 was found to be co-localized with MTAs from five different studies. Similarly, MQTLs identified for different traits in different crops have been most recently validated with GWAS results, such as multiple disease resistance (Pal et al., 2022; Saini et al., 2022a) and grain yield and related traits (Saini et al., 2022c). Further, genes *Phvul.004G005800* and *Phvul.004G006800* that were found to be associated with MQTL4.1 in this study were validated by two different GWA studies (Zuiderveen et al., 2016; Almeida et al., 2021). Similarly, the gene *Phvul.001G141000* available from MQTL1.3 was validated in two earlier GWA studies (Perseguini et al., 2016; VazBisneta and Gonçalves-Vidigal, 2020). These genes have been shown to participate actively in a variety of biological processes in order to promote disease resistance in common beans (Wu et al., 2017; VazBisneta and Gonçalves-Vidigal, 2020). *Phvul.004G012801* and *Phvul.001G128200.1*, for example, encode NBS-LRR domain containing proteins that are widely known to provide disease resistance. Overall, these GWAS-validated MQTLs may prove more useful for breeding programs as they are not believed to be influenced by genetic background. Further, the identification of these GWAS validated MQTLs provided a basis for accurately mining CGs affecting anthracnose resistance in common bean.

## Conclusion

Meta-analysis of QTLs and functional analysis enabled us to uncover the intricate genetic architecture underlying anthracnose disease resistance in common beans. Identified MQTLs each involving different QTLs that regulate anthracnose disease resistance in common beans may help in marker-assisted breeding programs. Four most promising MQTLs including “MQTL1.3,” “MQTL4.1,” “MQTL4.2” and “MQTL1.2” were selected and recommended to be exploited in breeding programs. Breeders from all over the world can employ either of these most promising MQTLs and identified CGs to improve the anthracnose disease resistance in common beans.

## Data availability statement

The original contributions presented in this study are included in the article/Supplementary material, further inquiries can be directed to the corresponding authors.

## Author contributions

All authors contributed to the study conception and design. RRM provided the conceptualization of the manuscript. SS, NC, VB, WAD, and DS contributed to material preparation and data collection and analysis. RRM and MAK performed proofreading. SS wrote the first draft of the manuscript. AKP and RKV contributed to review and editing. All authors commented on previous versions of the manuscript and read and approved the final manuscript.

## Footnotes

1
https://scholar.google.com/

2
https://pubmed.ncbi.nlm.nih.gov/

3
https://phytozome-next.jgi.doe.gov/
