# Supplementary material for: Delineating meta-quantitative trait loci for anthracnose resistance in common bean (Phaseolus vulgaris L.)
Source: Front Plant Sci. 2022 Aug 25;13:966339. doi: 10.3389/fpls.2022.966339 (PMC9453441; doi:10.3389/fpls.2022.966339)
Supplement: Supplementary file 2 [file Image_1.pdf]

Delineating meta-quantitative trait loci for anthracnose resistance in common bean (*Phaseolus vulgaris* L .)

Frontiers in Plant Science

Safoora Shafi[1], Dinesh Kumar Saini[2], Mohd Anwar Khan[1], Vanya Bawa[3], Neeraj Choudhary[3], Waseem Ali Dar[4], Arun K. Pandey[5], Rajeev Kumar Varshney[6]* andReyazul Rouf Mir[1]*

[1]Division of Genetics and Plant Breeding, Faculty of Agriculture, SKUAST-Kashmir, Wadura-193201, India

[2]Department of Plant Breeding and Genetics, Punjab Agricultural University, Ludhiana, Punjab, India

[3]Division of Genetics & Plant Breeding, Faculty of Agriculture, SKUAST-Jammu, Chatha-180009, J&K, India

[4]Mountain Agriculture Research and Extension Station, SKUAST-Kashmir, Gurez, Bandipora, Kashmir

[5]College of Life Sciences, China Jiliang University, Hangzhou 310018, China

[6]Agricultural Biotechnology Centre, Centre for Crop & Food Innovation, Food Futures Institute, Murdoch University, Murdoch, WA 6150, Australia

***Corresponding author email address:** Rajeev.Varshney@murdoch.edu.au; **rrmir@skuastkashmir.ac.in**

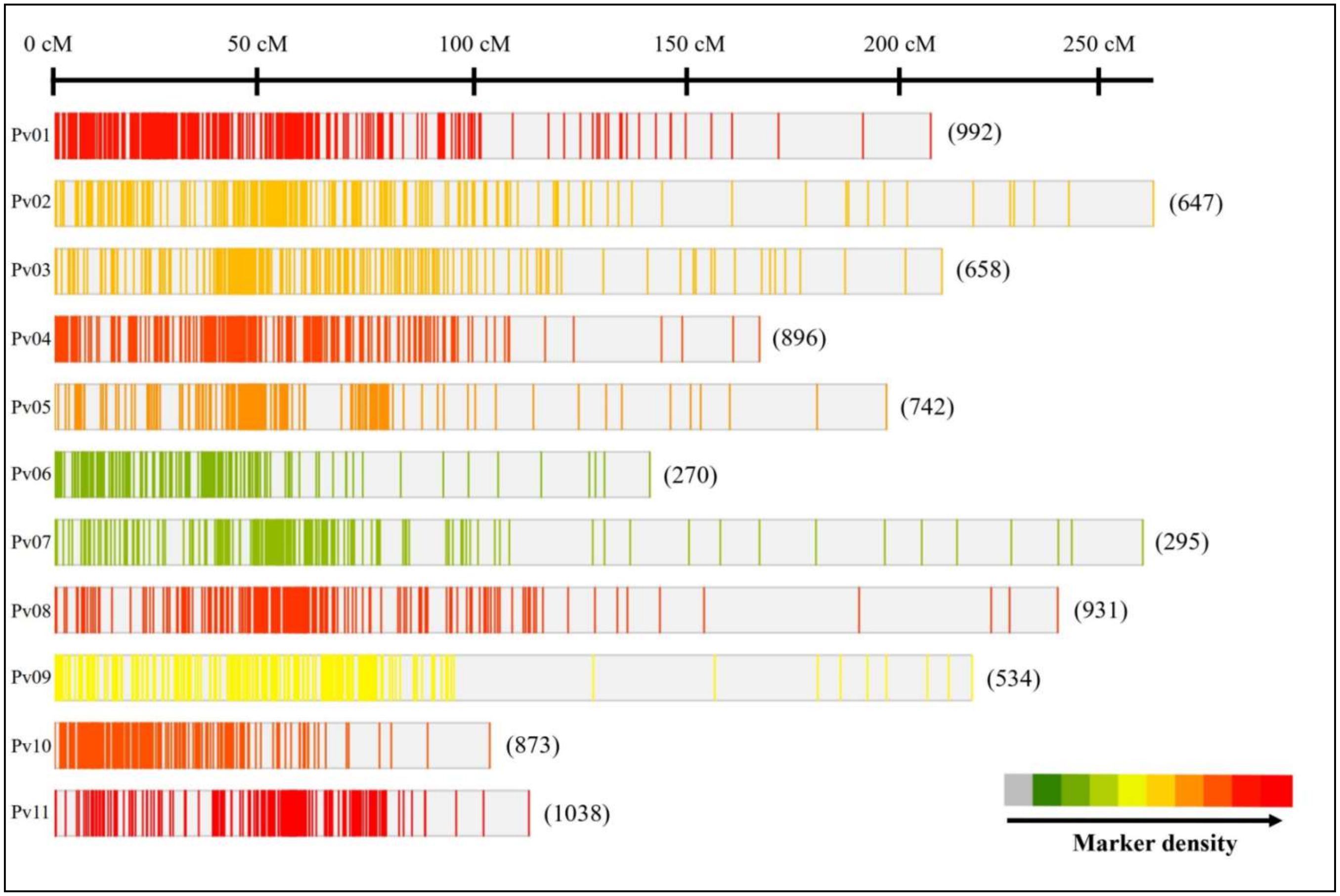

Fig. S1: Distribution of markers on the different chromosomes of the consensus map used during the present study in the meta-QTL analysis (number of markers mapped on each of the chromosomes are given in the parenthesis).
